# Compression and tension variably alter Osteoprotegerin expression via miR-3198 in periodontal ligament cells

**DOI:** 10.1186/s12860-019-0187-2

**Published:** 2019-04-04

**Authors:** Hiroyuki Kanzaki, Satoshi Wada, Yuuki Yamaguchi, Yuta Katsumata, Kanako Itohiya, Sari Fukaya, Yutaka Miyamoto, Tsuyoshi Narimiya, Koji Noda, Yoshiki Nakamura

**Affiliations:** 1 Tohoku University Hospital, Maxillo-oral Disorders, Sendai, Japan; 2 Department of Orthodontics, School of Dental Medicine, Tsurumi University, 2-1-3 Tsurumi, Tsurumi-ku, Yokohama, Kanagawa pref 230-8501 Japan

**Keywords:** MicroRNA, Osteoprotegerin, Orthodontic tooth movement, miR-3198, Mechanical stresses

## Abstract

**Background:**

Osteoclasts play a critical role in bone resorption due to orthodontic tooth movement (OTM). In OTM, a force is exerted on the tooth, creating compression of the periodontal ligament (PDL) on one side of the tooth, and tension on the other side. In response to these mechanical stresses, the balance of receptor activator of nuclear-factor kappa-B ligand (RANKL) and osteoprotegerin (OPG) shifts to stimulate osteoclastogenesis. However, the mechanism of OPG expression in PDL cells under different mechanical stresses remains unclear. We hypothesized that compression and tension induce different microRNA (miRNA) expression profiles, which account for the difference in OPG expression in PDL cells.

To study miRNA expression profiles resulting from OTM, compression force (2 g/cm^2^) or tension force (15% elongation) was applied to immortalized human PDL (HPL) cells for 24 h, and miRNA extracted. The miRNA expression in each sample was analyzed using a human miRNA microarray, and the changes of miRNA expression were confirmed by real-time RT-PCR. In addition, miR-3198 mimic and inhibitor were transfected into HPL cells, and OPG expression and production assessed.

**Results:**

We found that certain miRNAs were expressed differentially under compression and tension. For instance, we observed that miR-572, − 663, − 575, − 3679-5p, UL70-3p, and − 3198 were upregulated only by compression. Real-time RT-PCR confirmed that compression induced miR-3198 expression, but tension reduced it, in HPL cells. Consistent with previous reports, OPG expression was reduced by compression and induced by tension, though RANKL was induced by both compression and tension. OPG expression was upregulated by miR-3198 inhibitor, and was reduced by miR-3198 mimic, in HPL cells. We observed that miR-3198 inhibitor rescued the compression-mediated downregulation of OPG. On the other hand, miR-3198 mimic reduced OPG expression under tension. However, RANKL expression was not affected by miR-3198 inhibitor or mimic.

**Conclusions:**

We conclude that miR-3198 is upregulated by compression and is downregulated by tension, suggesting that miR-3198 downregulates OPG expression in response to mechanical stress.

## Background

Orthodontic tooth movement (OTM) describes the orchestrated responses of periodontal tissues in response to physical force. During OTM, site-specific bone metabolisms take place simultaneously: osteoclastic bone resorption in the compression zone and osteoblastic bone formation in the tension zone of periodontal ligament (PDL) [1–3]. Osteoclastogenesis is primarily regulated by receptor activator of nuclear-factor kappa-B ligand (RANKL) [4]. RANKL signaling is inhibited by osteoprotegerin (OPG), and the balance between RANKL and OPG contributes to the regulation of bone resorption [5]. The relationship between the RANKL/OPG ratio and progression of OTM has been extensively studied. Compression induces RANKL expression [6–9] and reduces OPG expression [10, 11] in PDL cells, thereby increasing the RANKL/OPG ratio, and favoring RANKL-mediated osteoclastogenesis. Conversely, tension increases OPG expression in PDL cells both in vivo [12, 13] and in vitro [14–17], which in turn inhibits RANKL. However, the mechanism regulating OPG expression in PDL cells under different mechanical stresses remains unclear.

The relationship between mechano-sensing and microRNA (miRNA) expression has recently become clearer. It is now understood that miRNAs in vascular endothelial cells play an essential role in shear stress-regulated endothelial responses [18]. Mechanical stretch regulates microRNA expression in C2C12 myoblasts [19]. Furthermore, mechanical stress can induce expression of miRNAs that modulate the expression of osteogenic and bone resorption factors, thus effecting bone remodeling due to mechanical stresses [20]. These studies suggest miRNAs might play a role in the regulation of differential OPG expression in PDL cells under compression and tension.

We hypothesized that compression and tension induce different miRNA expression profiles, resulting in differential OPG expression in PDL cells. To test this hypothesis, in the present study we used miRNA microarrays to examine miRNA expression profiles of cultured PDL cells under compression and tension. We identified several miRNAs that were differentially regulated during compression and tension, and, using target prediction databases, identified OPG as a potential target for the miRNA *miR-3198*. We found *miR-3198* was upregulated by compression and downregulated by tension. Augmentation and attenuation of *miR-3198* by a miRNA mimic and an inhibitor, respectively, revealed that *OPG* expression was downregulated by *miR-3198*.

Thus, we show here that compression and tension differentially regulate miRNA expression. Importantly, we found that *miR-3198*, which was induced by compression and reduced by tension, downregulates *OPG* expression in PDL cells.

## Results

### miRNA expression is mediated by mechanical stress

We examined miRNA expression in human PDL (HPL) cells in three different groups (control, compression, and tension) using a microarray. The top 20 differentially expressed miRNAs between the control and compression groups, the control and tension groups, and the tension and compression groups were identified (Table 1). Some miRNAs, such as *miR-1268*, *− 3648*, *−642b*, and *-135a*, were upregulated in both the compression and tension groups compared with the control group. The data suggest these miRNAs are upregulated by any type of mechanical stress. miRNAs such as *miR-572*, *− 663*, *− 575*, *− 3679-5p*, *UL70-3p*, and *− 3198* were upregulated in the compression group compared with both the control group and tension group, suggesting that these miRNAs are specifically upregulated by compression. miRNAs upregulated in the tension group relative to the control group included *miR-376a*. These results suggest that some miRNAs are upregulated in response to any type of mechanical stress, whilst other miRNAs are upregulated only in response to a specific type of mechanical stress.

**Table 1:** microarray analysis for miRNA expression

control VS Compression	Log2 Ratio	control VS Tension	Log2 Ratio	Tension VS Compression	Log2 Ratio
hsa-miR-1268	9.94	hsa-miR-3648	7.37	hsa-miR-4299	11.10
hsa-miR-572	7.73	hsa-miR-1268	6.57	hsa-miR-572	7.73
hsa-miR-663	7.60	hsa-miR-642b	6.31	hsa-miR-663	7.60
hsa-miR-3648	7.21	hsa-miR-135a	5.35	hsa-miR-575	6.87
hsa-miR-575	6.87	hsa-miR-376a	5.21	hsa-miR-3679-5p	6.68
hsa-miR-3679-5p	6.68	hsa-miR-4271	1.64	hcmv-miR-UL70-3p	6.57
hsa-miR-642b	6.64	hsa-miR-136	1.47	hsa-miR-3198	6.56
hcmv-miR-UL70-3p	6.57	hsa-miR-29b	1.36	hsa-miR-1305	6.47
hsa-miR-3198	6.56	hsa-miR-3663-3p	1.36	hsa-miR-1225-3p	6.31
hsa-miR-1305	6.47	hsv1-miR-H18	1.28	hsa-miR-125a-3p	6.16
hsa-miR-1225-3p	6.31	hsa-miR-3656	−1.04	hsa-miR-1246	6.14
hsa-miR-125a-3p	6.16	ebv-miR-BART13	−1.16	hsv1-miR-H17	5.88
hsa-miR-1246	6.14	hsa-miR-145	−1.28	hsa-miR-140-3p	5.79
hsv1-miR-H17	5.88	hsa-miR-181b	−1.35	hsa-miR-155	5.75
hsa-miR-654-5p	5.48	hsa-miR-181a-2	−1.42	hsa-miR-654-5p	5.48
hsa-miR-135a	5.43	hsa-miR-503	−4.99	hsa-miR-129-3p	5.37
hsa-miR-129-3p	5.37	hsa-miR-425	−5.43	hsa-miR-874	5.36
hsa-miR-874	5.36	hsa-miR-425	−5.61	hsa-miR-425	5.26
hsa-miR-4299	5.32	hsa-miR-4299	−5.78	hsv1-miR-H7	5.26
hsv1-miR-H7	5.26	hsa-miR-155	−5.87	hsa-miR-485-3p	5.26

### miRNAs targeting OPG are regulated by mechanical stress

Since OPG expression is regulated by mechanical stress in OTM, we investigated whether miRNAs upregulated by mechanical stress were also miRNAs predicted to target *OPG*. miRNAs that target *OPG* were predicted by two databases (Table 2). *miR-1207* was on neither list; therefore an *miR-1207* mimic and inhibitor were used as negative controls. We identified miR-3198 on both of the lists, raising the possibility that compression-induced miR-3198 downregulates OPG expression.

**Table 2:** microRNAs which target OPG (TNFRSF11b)

microRNA.org				miRDB.org			
RANK and miRNA				RANK and miRNA			
1	hsa-miR-3163	26	hsa-miR-135a	1	hsa-let-7f-2-3p	26	hsa-miR-6870-3p
2	hsa-miR-586	27	hsa-miR-135b	2	hsa-miR-1185-1-3p	27	hsa-miR-936
3	hsa-miR-633	28	hsa-miR-200b	3	hsa-miR-1185-2-3p	28	hsa-miR-5692a
4	hsa-miR-656	29	hsa-miR-590-5p	4	hsa-miR-4262	29	hsa-miR-145-5p
5	hsa-miR-130b	30	hsa-miR-21	5	hsa-miR-3163	30	hsa-miR-5195-3p
6	hsa-miR-548c-3p	31	hsa-miR-4255	6	hsa-miR-892c-5p	31	hsa-let-7c-3p
7	hsa-miR-590-3p	32	hsa-miR-4309	7	hsa-miR-5584-5p	32	hsa-miR-216a-5p
8	hsa-miR-577	*33*	**hsa-miR-3198**	8	hsa-miR-4729	33	hsa-miR-4753-3p
9	hsa-miR-579	34	hsa-miR-2054	9	hsa-miR-181a-5p	34	hsa-miR-590-5p
10	hsa-miR-576-5p	35	hsa-miR-936	10	hsa-miR-181c-5p	35	hsa-miR-3160-5p
11	hsa-miR-429	36	hsa-miR-380	11	hsa-miR-181d-5p	36	hsa-miR-429
12	hsa-miR-488	37	hsa-miR-3172	12	hsa-miR-181b-5p	37	hsa-miR-200b-3p
13	hsa-miR-4262	38	hsa-miR-376a	13	hsa-miR-3942-3p	38	hsa-miR-200c-3p
14	hsa-miR-181a	39	hsa-miR-376b	14	hsa-miR-4766-3p	39	hsa-miR-765
15	hsa-miR-181b	40	hsa-miR-145	15	hsa-miR-4668-5p	40	hsa-miR-5582-5p
16	hsa-miR-181c	41	hsa-miR-193b	16	hsa-miR-3942-5p	41	hsa-miR-629-5p
17	hsa-miR-181d	42	hsa-miR-4307	17	hsa-miR-4294	42	hsa-miR-6892-5p
18	hsa-miR-1283	43	hsa-miR-765	18	hsa-miR-4703-5p	43	hsa-miR-577
19	hsa-let-7f-2	44	hsa-miR-374a	19	hsa-miR-6501-3p	44	hsa-miR-579-3p
20	hsa-miR-889	45	hsa-miR-29b-2	20	hsa-miR-506-3p	45	hsa-miR-183-3p
21	hsa-miR-4294	46	hsa-miR-570	21	hsa-miR-124-3p	46	hsa-miR-6074
22	hsa-miR-187	47	hsa-miR-188-3p	22	hsa-miR-3662	47	**hsa-miR-3198**
23	hsa-miR-200c	48	hsa-miR-222	23	hsa-miR-130b-5p	48	hsa-miR-513b-3p
24	hsa-miR-506	49	hsa-miR-3170	24	hsa-miR-193b-5p	49	hsa-miR-7109-3p
25	hsa-miR-124	50	hsa-miR-1323	25	hsa-miR-5582-3p	50	hsa-miR-4309

miR-3198, which is found in the table 1, is indicated by boldface

### Expression of *miR-3198* and *OPG* was regulated differentially in compression and tension

**Fig. 1:**
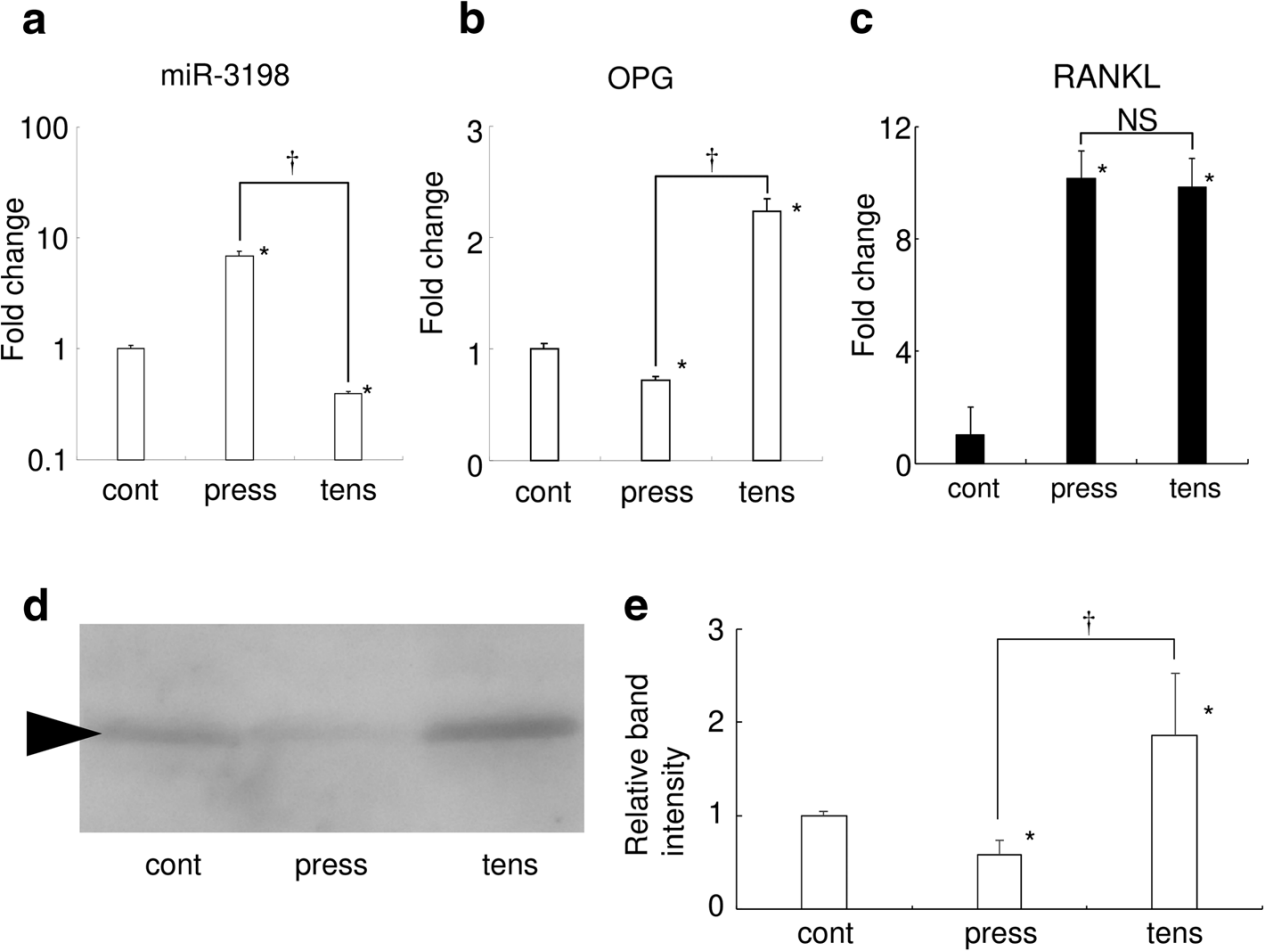
miR-3198 and *OPG* were regulated differentially by compression and tension. The results of real-time RT-PCR analysis for miR-3198 (**a**), *OPG* (**b**), and *RANKL* (**c**) expression in HPL cells are shown. *n* = 3. Biological triplicated. Fold change from the control is displayed, with *P* < 0.05 versus control indicated by * and *P* < 0.05 between samples indicated by †. NS: not significant difference between the groups. Cont: control, press: compression, tens: tension. **d** Representative image of the western blotting (biological triplicate) for OPG is shown. **e** Relative band intensity of the western blotting for OPG. *: *P* < 0.05 versus control. †: *P* < 0.05 between the groups

Real-time RT-PCR analysis confirmed the effects of *− 3198* due to mechanical stress. Compression induced miR-3198 expression in HPL cells, whereas tension reduced miR-3198 expression (Fig. 1a). These results were consistent with the miRNA array analysis. *OPG* expression was reduced by compression but was induced by tension (Fig. 1b). *RANKL* expression was increased by both compression and tension (Fig. 1c). Western blotting revealed that compression reduced OPG protein level in the cultured HPL cells (Fig. 1d and e). In addition, tension increased OPG protein level in the cultured HPL cells. These results suggest that miR-3198 downregulates OPG expression in response to mechanical stress.

### miR-3198 gain-of-function and loss-of-function experiments

**Fig. 2:**
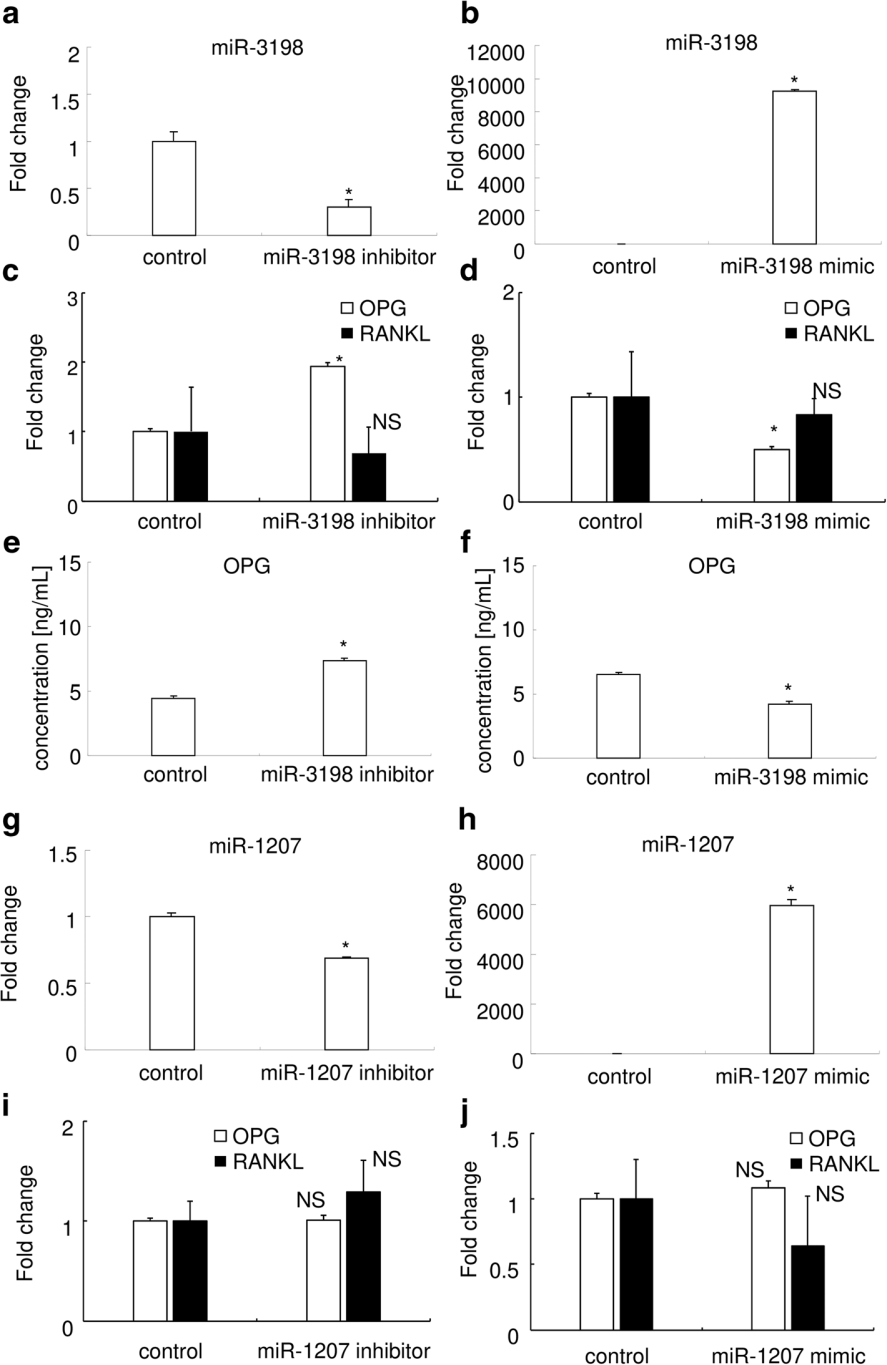
miR-3198 gain-of-function and loss-of-function experiments. Results of real-time RT-PCR analysis for miR-3198 (**a**, **b**), *OPG* and *RANKL* (**c**, **d**) expression in HPL cells after transfection of miR-3198 inhibitor (**a**, **c**) and miR-3198 mimic (**b**, **d**). *n* = 3. Biological triplicated. Fold change from the control is shown. Open bar indicates the fold change of OPG expression, and close bar indicates that of RANKL expression (**c**, **d**). The concentrations of OPG as measured by ELISA after transfection of miR-3198 inhibitor (**e**) and miR-3198 mimic (**f**) are shown (*n* = 3). Results of real-time PCR analysis for miR-1207 (**g**, **h**), *OPG* and *RANKL* (**i**, **j**) expression in HPL cells under the transfection of miR-1207 inhibitor (**g**, **i**) and miR-1207 mimic (**h**, **j**). *n* = 3. Biological triplicated. Fold changes from the control are shown. * indicates *P* < 0.05 versus control, and NS indicating there was no significant difference between samples

We further examined the relationship between miR-3198 and OPG expressions through gain- and loss-of-function experiments. We found that transfection of miR-3198 inhibitor in HPL cells reduced miR-3198 expression (Fig. 2a), whereas transfection of miR-3198 mimic upregulated miR-3198 expression (Fig. 2b). Similarly, *OPG* mRNA expression was induced by miR-3198 inhibitor (Fig. 2c) and reduced by miR-3198 mimic (Fig. 2d). *RANKL* mRNA expression was stable irrespective of the transfection of miR-3198 inhibitor or mimic. OPG protein levels were also increased by miR-3198 inhibitor (Fig. 2e) and decreased by miR-3198 mimic (Fig. 2f).

To clarify whether these phenomena were dependent on miR-3198 specifically, we examined the role of miR-1207, which was not predicted to target OPG. We found that the transfection of miR-1207 inhibitor reduced miR-1207 expression in HPL cells (Fig. 2g), and the transfection of miR-1207 mimic upregulated miR-1207 expression (Fig. 2h), consistent with the results of the miR-3198 inhibitor and mimic experiment. *OPG* and *RANKL* expression were stable regardless of the transfection of miR-1207 inhibitor or mimic (Fig. 2i and j).

These results suggest that mechanical stress-induced miR-3198 downregulates OPG expression in HPL cells.

### miR-3198 regulates mechanical stress-mediated OPG expression

**Fig. 3:**
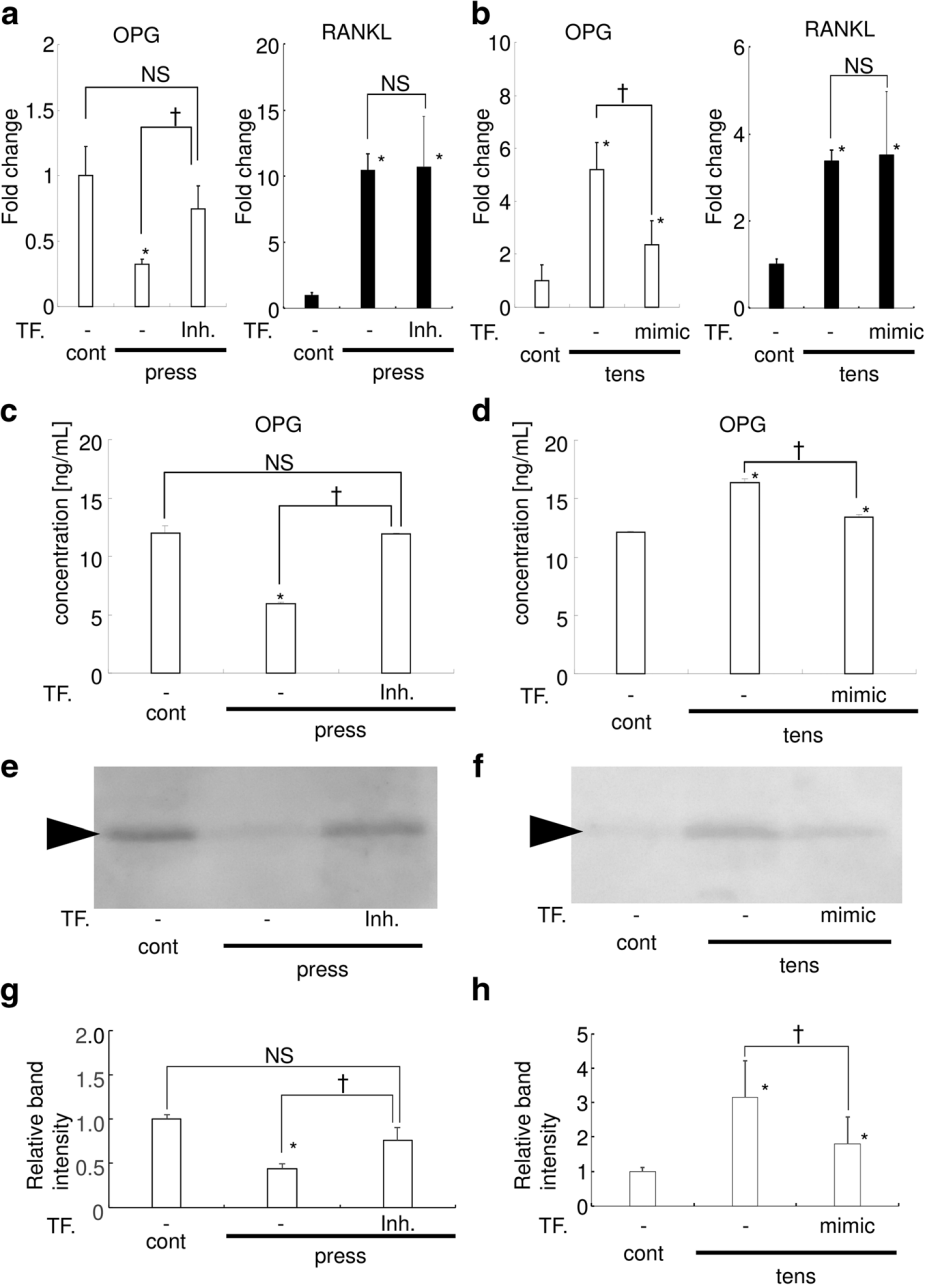
miR-3198 regulates the mechanical stress-mediated change of OPG expression. Results of real-time RT-PCR analysis for *OPG* and *RANKL* expression in HPL cells in the compression (**a**) and tension (**b**) experiments. *n* = 3. Biological triplicated. Fold change from the control are shown. Cont, control; Inh, transfection of miR-3198 inhibitor; Mimic, transfection of miR-3198 mimic; press, compression; tens, tension; TF, transfection. Also shown are the OPG concentrations measured by ELISA in the compression (**c**) and tension (**d**) experiments (*n* = 3). * indicates *P* < 0.05 versus control. † indicates *P* < 0.05 between samples. NS indicates there was no significant difference between samples. **e** and **f** Representative image of the western blotting for OPG was shown. **g** and **h** Relative band intensity of the western blotting for OPG. *: *P* < 0.05 versus control. †: *P* < 0.05 between the groups. NS, no significant difference between the samples

Finally, we examined the role of miR-3198 in the regulation of mechanical stress-mediated OPG expression. We found that *OPG* expression was reduced by compression, although transfection of miR-3198 inhibitor rescued the compression-mediated downregulation of *OPG* (Fig. 3a). In addition, there was no significant difference between the control and the compression + miR-3198 inhibitor groups, indicating that miR-3198 plays a role in the regulation of *OPG* expression under compression. *RANKL* mRNA expression was upregulated by compression, and it was stable irrespective of the transfection of miR-3198 inhibitor.

Conversely, we found that under tension, augmentation of miR-3198 expression by miR-3198 mimic reduced *OPG* expression (Fig. 3b). There was a significant difference in the OPG expression levels between the control and the tension + miR-3198 mimic (*P* = 0.03). *RANKL* mRNA expression was upregulated by tension, and it was stable irrespective of the transfection of miR-3198 mimic.

Consistent with results of real-time PCR, OPG quantification by ELISA revealed that compression reduced OPG protein levels (Fig. 3c), whereas miR-3198 inhibitor prevented the compression-mediated reduction of OPG. In addition, tension upregulated OPG production (Fig. 3d). Similarly, we found that miR-3198 mimic significantly reduced the tension-mediated increase in OPG production.

Western blotting for OPG also revealed that the miR-3198 inhibitor prevented the compression-mediated reduction of OPG (Fig. 3e and g). On the other hand, miR-3198 mimic significantly reduced the tension-mediated increase in OPG production (Fig. 3f and h). These results indicate that miR-3198 downregulates OPG expression in HPL cells under mechanical stress.

## Discussion

Osteoclastic bone resorption is tightly regulated by RANKL [4] in the periodontal ligament during OTM [7, 10]. Conversely, OPG, the decoy receptor to RANKL, inhibits osteoclastogenesis [21]. The RANKL/OPG ratio increases at the compression zone of the PDL during OTM [10]. In vitro experiments have revealed that compression increases the RANKL/OPG ratio in PDL cells [9, 22–24]. On the other hand, tension decreases RANKL/OPG ratio in PDL cells, mainly by the induction of OPG expression [14–17]. Generally, OPG expression in the PDL cells usually increases under tension and decreases under compression. Though our previous report [15] and this study revealed upregulation of RANKL by tension, induction of OPG by tension predominates over RANKL, resulting in a low RANKL/OPG ratio and inactive osteoclastogenesis.

In this study, we examined whether miRNAs are involved in site-specific changes in OPG expression during OTM. We found that miRNAs were differentially regulated by compression and tension. In particular, miR-3198 was upregulated by compression and downregulated by tension. Furthermore, we found that miR-3198 regulates OPG expression in response to mechanical stresses, which is consistent with phenomena observed in the PDL during OTM; namely, osteoclastic bone resorption in the compression zone and osteoblastic bone formation in the tension zone of the PDL [3]. On the other hand, RANKL expression was not affected by miR-3198. Our present results indicate that tension-induced OPG expression is reduced by the overexpression of miR-3198 mimic, although we did find a significant difference in OPG expression between control and tension + miR-3198 mimic groups. Considering that miR-3198 plays a role in the regulation of OPG expression under different mechanical stresses, epigenetic regulation such as methylation of miR-3198 and mutation of miR-3198 would interfere the mechanical stress-mediated miR-3198 expression followed by the difference in OPG expression, which regulates the alveolar bone resorption during orthodontic tooth movement. Further studies will shed light on the importance of miR-3198 on the regulation of the alveolar bone metabolism during orthodontic tooth movement.

Regarding the relationship between OTM and miRNA, Chen et al. reported that miR-21 deficiency attenuated OTM via inhibition of alveolar bone resorption on both the compressive and tensile sides [25]. In addition, Chang et al. reported the role of miRNA in tension force-induced bone formation [26]. They concluded that miR-195-5p, miR-424-5p, miR-1297, miR-3607-5p, miR-145-5p, miR-4328, and miR-224-5p were core miRNAs of tension force-induced bone formation. Within these miRNA, no miRNAs were changed by mechanical stresses in our experiment, except miR-145. We presumed that the difference in observed miRNAs between Chang’s study and ours would be due to the different time points assessed (at 72 h in Chang’s paper, and at 24 h in ours). We wanted to explore the early response of PDL cells against mechanical stress via miRNA, and examined only at 24 h, the timing the other researchers tested at [27, 28]. Exploration of the time course change of each miRNA would be useful to clarify this. Liu et al. reported that miR-503-5p functions as a mechano-sensitive miRNA and inhibits bone marrow stromal cell osteogenic differentiation subjected to mechanical stretch and bone formation in OTM tension sides [29]. Chen et al. reported that cyclic stretch decreased, and compression increased, the expression of miR-29 in PDL cells, which directly interacts with *Col1a1*, *Col3a1* and *Col5a1* [30]. These studies reveal the relationship between mechanical stress-mediated miRNA expression and bone formation or tissue remodeling. However, the effects of miRNA on the RANKL/OPG ratio during OTM were unclear until now.

Regarding the regulation of OPG expression by miRNAs, miRs-21 [31, 32], − 145 [33], −146a [34], − 150 [35], and − 200 [36] have been reported to regulate OPG expression. Among them, miRs-21, − 145, and − 200 were thought to be direct regulators of OPG expression in the databases of miRDB.org and microRNA.org. Therefore, we presumed that the refinement of candidate miRNAs using the available databases was a sufficiently accurate method to choose candidates.

We found that miR-3198 plays a role in the regulation of the mechanical stress-mediated OPG expression, although the reciprocal regulatory mechanism of miR-3198 by compression and tension remains unclear. Some mechanical stresses induce differential intracellular signaling systems, such as G-proteins, calcium signaling, MAPK signaling, and nitric oxide signaling [37]. Further studies are needed to clarify the regulatory mechanism of miR-3198 by compression and tension. miR-3198 was identified in human tumor breast tissue [38], and is on 22q11.21 of the genome. It is important to confirm that miR-3198 downregulates OPG expression by mechanical stress in animal models. However, there is no orthologue of miR-3198 in mice or rats, which makes it difficult to conduct such experiments. Nevertheless, further confirmatory experiments are required.

## Conclusions

In conclusion, we found that miRNAs were differentially regulated by compression and tension in PDL cells. Furthermore, miR-3198 downregulates OPG expression in PDL cells in response to mechanical stress.

## Methods

### Cells

Human immortalized periodontal ligament cell lines (HPL cells) were received from the University of Hiroshima, Hiroshima, Japan, where they were originally established [39]. HPL cells were cultured in alpha modified Eagle’s medium (Wako Pure Chemical, Osaka, Japan) containing 10% fetal bovine serum (Thermo Fisher Scientific, Waltham, MA) and supplemented with penicillin (100 U/mL) and streptomycin (100 μg/mL). All cells were cultured at 37 °C in a 5% CO_2_ incubator.

### Application of mechanical stress

Compressive force was applied to the HPL cells using a glass cylinder, as described elsewhere [9]. Briefly, a glass cylinder was placed over a confluent cell layer in the well of a 6-well plate. HPL cells were subjected to 2 g/cm^2^ of compressive force for 24 h. Cyclical tensile force was applied to HPL cells with a Flexercell Strain-Unit (Flexcell Corp., Hillsborough, NC, USA), as described elsewhere [15]. Briefly, PDL cells were pre-cultured in flexible-bottomed culture plates coated with type I collagen until confluent. The culture plates were then set on the rubber gasket of the Flexercell Strain Unit, and PDL cells were subjected to cyclical tensile force (15% elongation, 1 s stretch/1 s relaxation) for 24 h.

### miRNA and RNA extraction

miRNA and RNA were extracted separately from HPL cells using the Nucleospin miRNA isolation kit (Macherey-Nagel, Düren, Germany), according to the manufacturer’s instructions.

### miRNA array analysis

The quality of the extracted miRNAs was examined by an Agilent 2100 Bioanalyser (Agilent Technologies, Santa Clara, CA). RNA integrity numbers ranged from 8.7 to 9.5. miRNA expression in each sample was analyzed using a SurePrint G3 Human miRNA microarray 8 × 60 K miRBase 16.0 (Agilent Technologies), according to the manufacturer’s instructions.

### Database analysis for miRNAs target prediction

To identify candidate miRNA which targeted OPG, two target prediction databases, miRDB.org [40] and microRNA.org [41], were used. Candidate miRNAs were queried using “OPG” or “TNFRSF11B” as keywords.

### Reverse transcription (RT) and real-time RT-PCR analysis

Isolated miRNA (2 μg each) were reverse-transcribed (RT) with the miScript II RT kit (Qiagen, Germantown, MD), according to the manufacturer’s instructions. After reverse transcription, cDNA samples were diluted 5× with TE buffer. Real-time RT-PCR was performed using the miScript SYBR green PCR kit (Qiagen). The following PCR primers were used for the detection of miRNA: miR-1207 (MIMAT0005871), miR-3198 (MIMAT0015083), and *RNU6B*. Fold change of miR-3198 expression relative to the control was calculated by the Δ-Δ Ct method with *RNU6B* as a reference gene. Isolated RNA (500 ng) was reverse-transcribed using the iScript cDNA-Supermix (Bio-Rad, Hercules, CA, USA), according to the manufacturer’s instruction. After reverse transcription, cDNA samples were diluted 5× with TE buffer. Real-time RT-PCR was performed using the SsoFast EvaGreen-Supermix (Bio-Rad). PCR primers used for the experiments were human *OPG* (forward, 5′-AAGGGCGCTACCTTGAGATAG-3′; reverse, 5′-GCAAACTGTATTTCGCTCTGGG-3′), human *RANKL* (forward, 5′-CGTTGGATCACAGCACATCAG-3′; reverse, 5′-GCTCCTCTTGGCCAGATCTAAC-3′), and *human ribosomal protein S18* (*RPS18*) (forward, 5′-GATGGGCGGCGGAAAATAG-3′; reverse, 5′-GCGTGGATTCTGCATAATGGT-3′). Fold changes of *OPG* and *RANKL* expression relative to the control were calculated by the Δ-Δ Ct method with *RPS18* as a reference gene.

### miR-3198 gain-of-function and loss-of-function experiments

To observe the influence of miR-3198 on OPG expression, miR-3198 mimic (Qiagen) and miR-3198 inhibitor (Qiagen) were transfected into HPL cells using the TransIT-TKO® transfection reagent (Mirus Bio LLC, Madison, WI), according to the manufacturer’s instructions. miR-1207 mimic (Qiagen) and miR-1207 inhibitor (Qiagen) were used as the negative control. miR mimic and miR inhibitor were used at a final concentration of 50 nM (stock concentration, 20 μM), according to the manufacturer’s recommendation. The expression of miR-3198 was observed at 24 h after transfection. In some experiments, mechanical stress was applied to transfected HPL cells, beginning 12 h after transfection.

### OPG ELISA

The concentration of OPG in the culture supernatant was measured using an OPG ELISA kit (Boster Biological Technology, Pleasanton, CA), according to the manufacturer’s instructions. Culture supernatants were diluted 5× prior to measurement.

### Western blotting for OPG

Culture supernatants were subjected to electrophoresis on TGX Precast gels (BioRad), proteins were transferred to a PVDF membrane, which was blocked with PVDF Blocking Reagent (Toyobo Co. Ltd., Osaka, Japan), then incubated with a rabbit IgG anti-OPG antibody (GeneTex, Irvine, CA, USA). After thorough washing with 0.5% Tween-20 in PBS (PBS-T), the membrane was incubated with a horseradish peroxidase-conjugated anti-rabbit IgG antibody (R&D Systems, Inc., Minneapolis, MN, USA). Chemiluminescence was produced by using Luminata-Forte (EMD Millipore, Billerica, MA) and detected with LumiCube (Liponics, Tokyo, Japan).

### Statistical analysis

All data are presented as mean ± SD. Comparisons between two groups were performed using Student’s t-test. Multiple comparisons were performed by using Tukey’s test. A *P*-value < 0.05 was considered statistically significant.

## Abbreviations

ELISA: Enzyme-linked immunoSorbent assay
HPL cells: Human immortalized periodontal ligament cell lines
miRNA: MicroRNA
OPG: Osteoprotegerin
OTM: Orthodontic tooth movement
PDL: Periodontal ligament
RANKL: Receptor activator of NF-κB ligand
RT-PCR: Reverse transcription polymerase chain reaction
TGF-beta: Transforming growth factor beta
